# A rapid point of care CC16 kit for screening of occupational silica dust exposed workers for early detection of silicosis/silico-tuberculosis

**DOI:** 10.1038/s41598-021-02392-y

**Published:** 2021-12-06

**Authors:** Shyam Sundar Nandi, Upendra P. Lambe, Kamalesh Sarkar, Sonali Sawant, Jagadish Deshpande

**Affiliations:** 1 ICMR-National Institute of Virology, (Mumbai unit), Indian Council of Medical Research, Haffkine Institute Compound, A. D. Marg, Parel, Mumbai, 400012 India; 2 ICMR-National Institute of Occupational Health (ICMR-NIOH) Meghani Nagar, Ahmedabad, 380016 Gujarat India

**Keywords:** Biotechnology, Health occupations

## Abstract

Silicosis is an irreversible, incurable and progressive occupational disease caused by prolonged exposure to crystalline-silica dust while working in the relevant industries. Conventionally diagnosis is done by chest radiology, often in an advanced stage as early symptoms often go unnoticed. Early detection and necessary intervention (secondary prevention) could be a realistic possible control strategy for controlling silicosis as no effective treatment is available to stop and/or reverse the pathological process. Additionally, these patients are also vulnerable to pulmonary tuberculosis, which often becomes difficult to treat and with uncertain treatment outcome. Considering India has a huge burden of silicosis and silico-tuberculosis, a rapid and inexpensive screening method was realized to be an urgent need for early detection of silicosis among silica dust exposed workers. Serum club cell protein 16 (CC16) is evidenced to be a useful proxy screening marker for early detection of silicosis as evidenced from the recent research work of ICMR-National Institute of Occupational Health (ICMR-NIOH), India. In this study a lateral-flow assay for semi-quantitative estimation of serum CC16 level was developed. The detection was performed using gold nanoparticles conjugated anti-CC16 monoclonal antibodies. A sum of 106 serum samples was tested to do the performance evaluation of the assay. A concentration of 6 ng/ml or less produced one band, 6.1–9 ng/ml produced two bands, while more than 9 ng/ml produced all the three bands at the test zone. The sensitivity of the assay was 100% while the specificity was 95%. This assay may be used as a sensitive tool for periodic screening of silica dust exposed vulnerable workers for early detection of silicosis in them.

## Introduction

Club cell protein 16 (CC16) is the most abundant protein in broncho-alveolar secretions. CC16 inherits an anti-inflammatory property in the lungs. Chronic Obstructive Pulmonary disease (COPD) caused due to exposure of lungs to smoke or any other particulate pollutants^1^. CC16 has a molecular mass of 16 kilo Dalton, and belongs to the secretory globin sub-family of proteins. CC16 is a homodimeric protein with identical 70-amino acid subunits linked in an antiparallel orientation by two disulfide bonds^2^.

The main source of CC16 is the Club cell (formerly known as Clara cell) which is a non-ciliated, non-mucous secreting club-shaped cell present mainly in distal bronchioles as well as basal cells found in large airways. The density of Club cells throughout the respiratory tract varies substantially between species. In humans, Club cells represent 22% in the respiratory bronchioles^3^. Other organs that contain few CC16-secreting cells are the prostate, ovaries, pancreas, mammary glands, and uterine endometrium^4,5^.

As per the literature data, many chronic pulmonary inflammatory diseases such as anthraco-silicosis, chronic obstructive pulmonary disease (COPD), asthma etc. cause depletion of CC16. In all types of cases, Club cells are degenerated and reduced in number resulting in decreased levels of CC16 in Broncho-alveolar lavage fluid (BALF) and serum. The anti-inflammatory and protective effect of CC16 on the airway epithelium is gone resulting in inflammation of lungs. In case of acute inflammatory conditions, the CC16 level increases temporarily and attempts in the repair of the airway epithelium. But in cases of chronic exposures, the CC16 levels reduce gradually resulting in the chronic inflammation which ultimately leads towards fibrosis of lungs.

Chronic silicosis, the commonest and widely prevalent form of silicosis, is an irreversible occupational ailment of the respiratory system caused by the invasion of lung tissue (parenchyma) due to continuous or intermittent inhalation of dusts consisting of crystalline silica or silicon dioxide of respirable size (less than 10µ in diameter) while working in the relevant industries. Individuals with various durations of exposure ranging from 2 to 15 years or more in the above-said industries like mines, stone quarry, agate, construction sites and non-metallic product manufacturing units for example refractory (articles with heat resistant ability), ceramic, glass, mica, and structural clay are more prone to silicosis^6^. These micro-particles get trapped in the interstitial lung collagen tissue resulting in fibrosis of the lung. Therefore, it becomes one of the major occupational health hazards for the silica dust exposed workers working in relevant industries. Regrettably, most of the silicosis cases remain undiagnosed or misdiagnosed at an early stage due to asymptomatic or mild symptomatic nature of the initial stage of the disease, lack of suitable biomarker for early detection, poor health-seeking behavior of the workers and poor occupational health care delivery service at the workplaces, particularly in unorganized sectors^7–9^.

Patients with silicosis are prone to pulmonary tuberculosis also called silico-tuberculosis, probably due to destruction of alveolar macrophages (declined lung immunity). Differential diagnosis is difficult unless the physician is aware of the occupational history of silica exposure, which is very subjective in nature. Also, it becomes very difficult to differentiate between silicotic nodules and tuberculous infiltration in radiography. There is a lifelong risk of tuberculosis in silicotic patients even after stopping silica dust exposure. Secondly anti-tubercular treatment outcome is known to be uncertain with higher chance of reinfection in silicotic patients^10^. Additionally, isolation of *Mycobacterium bacilli* from the sputum of silico-tuberculosis is difficult  as moderate to advanced stage silicotic fibrosis prevents discharge of the *Mycobacterium* in sputum, making the situation more difficult^11^. Hence, a suitable biomarker is urgently required for early detection of silicosis among silica dust exposed workers. Early detection using a suitable bio-marker will prevent generating moderate and advanced silicosis and its associated co-morbidities (silico-tuberculosis, renal disease, stroke, heart disease, lung cancer etc.) by minimizing further dust exposure^12,13^. Hence, countries with high burden of silicosis are in need of a suitable bio-marker at an affordable cost as silicosis is a neglected occupational disease with high disease burden and victims are often from the under-privileged communities.

As per rule, the diagnosis of silicosis needs to be confirmed by chest radiology following clinical examination along with a history of occupational exposure to silica dust for a varying period. X-ray of chest shows bi-lateral pathognomonic patchy nodular opacities in silicosis. Diagnosis is invariably made at a moderate or an advanced stage when nothing could be done to save or prolong the patients’ lives. Considering all, a suitable biomarker for early suspicion of silicosis may be a useful screening tool for secondary prevention of silicosis and silico-tuberculosis to protect these vulnerable workers.

A number of anti-inflammatory biomarkers for early diagnosis of silicosis have been attempted, but most of these were found to be non-specific and hence unsuitable for detection of lung related pathologies with certainty^14,15^. Club cell protein (CC16) is secreted by Club cells of broncho-alveolar epithelial tissue of the lung^16^ (Bernard et al., 1994). CC16 is proposed to be a peripheral marker of respiratory epithelial injury that protects the respiratory tract against oxidative stress-induced inflammation^17–19^ and passively diffuses in broncho-alveolar-blood barrier to plasma^20^. The serum concentration of CC16 can be used to decipher the degree of chronic respiratory tract injury at an early stage. Though the exact physiological mechanism of CC16 remains unknown, but evidences suggest significant reduction of CC16 in chronically silica dust-exposed workers with no change in respiratory symptoms, normal chest radiology and lung function tests indicates that serum CC16 could be an early asymptomatic detection tool for silicosis among silica-exposed population at risk^21^. Above principle has been kept in mind while developing a point of care, semi-quantitative CC16 kit as a screening tool for early detection of silicosis.

At present-day, the CC16 detection is performed with commercially available enzyme linked immunosorbent (ELISA) assays of clinical field application^22^ (Biovendor–Laboratorni) that requires to be imported from the foreign countries. The available commercial assays are very expensive and the cost cannot be afforded by the daily wage workers and/or by the health authority for mass use. Moreover, it requires expensive instrumentation that is available only in the large cities. Thus, there is a need for economical, user friendly and rapid detection devices and methods which do not require expensive instrumentation or specialized skills for testing and analysis. The purpose of developing this kit is to facilitate even for the remote rural health care workers for use of it in need with little training.

In this current study, we describe a Point of Care assay that can be particularly employed for semi-quantitative estimation of CC16 in human serum samples. This assay can be used periodically at regular intervals to assess the serum CC16 levels among workers with the history of silica dust exposure. This assay would give an idea about an estimated lung injury caused by silica dust exposure before advising for their radiological confirmation to arrive at a confirmed diagnosis. So, it may be considered as a proxy bio-marker as well as a screening tool for early detection of silicosis among silica dust exposed workers exclusively. It should not be used for assessing other lung diseases without further disease specific validation.

## Results

In this study, 106 serum samples were tested. A sum total of 68 samples were obtained from silica dust exposed workers and confirmed by chest x-ray and 38 samples were obtained from healthy individuals without silica dust exposure history.

### ELISA for the quantitation of CC16

The CC16 concentrations in serum samples were determined using a commercially available ELISA kit (Catalogue No. RD191022200 Biovendor – Laboratorni medicina a.s. Czech Republic). This ELISA was used as the reference method for comparative evaluation of the semi-quantitative lateral flow assay. The results of ELISA were interpreted by the standard manufacturer’s protocol (Biovendor – Laboratorni medicina a.s. Czech Republic). All the x-ray confirmed silicotic subjects had CC16 levels < 9 ng/ml and healthy controls had CC16 concentration > 9 ng/ml.

### Lateral flow assay

**Figure 1:**
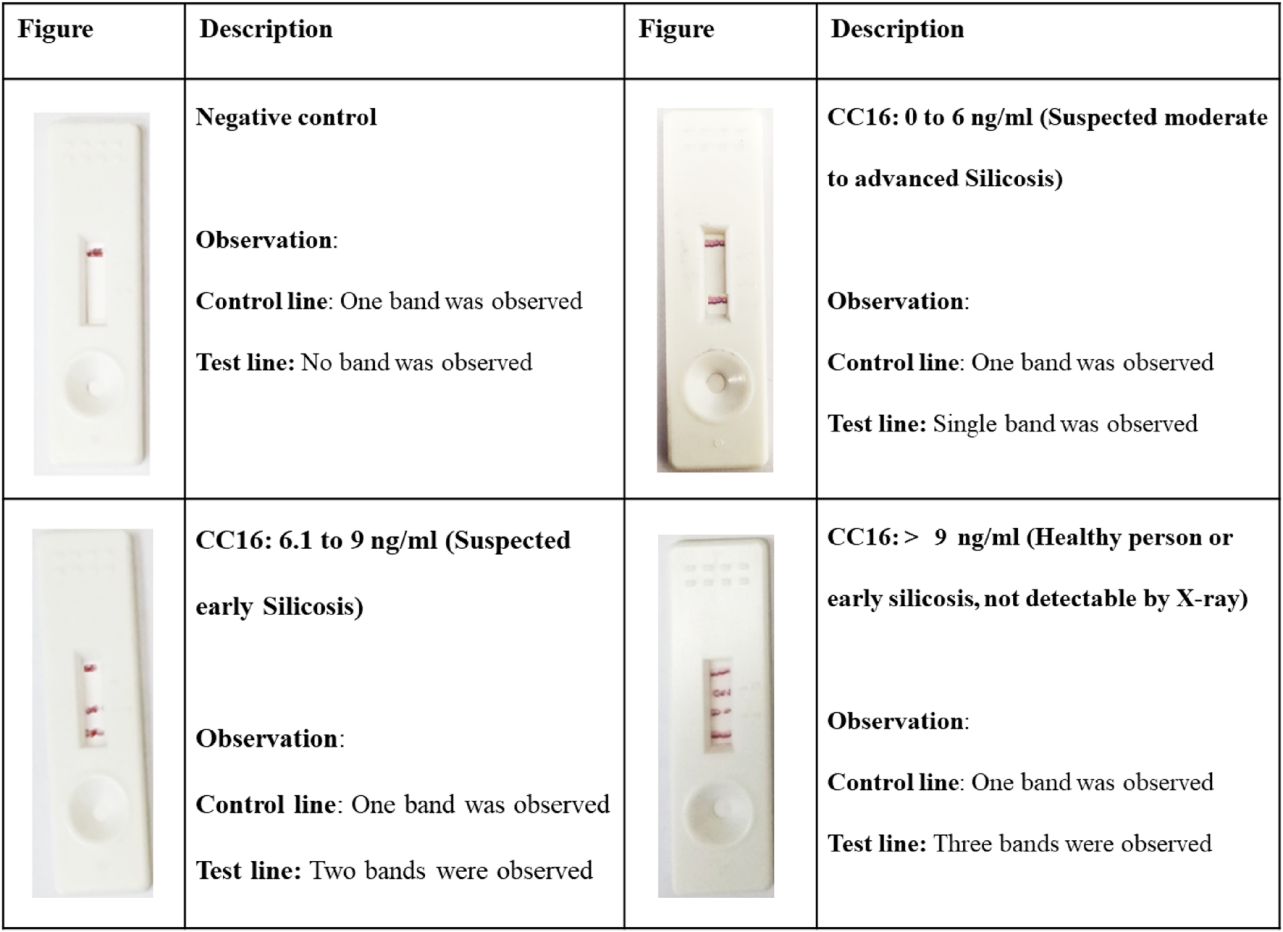
Detailed description for interpretation of results for semi-quantitative lateral flow assay for detection of CC16.

The interpretation of the results was done by checking the control line. The control line was indicative of valid reaction. If the control line does not appear, the reaction is invalid. If the serum CC16 concentration is 6 ng/ml or less, one red colored band was detected. If the serum CC16 concentration was in the range of 6.1– 9 ng/ml then the assay produced two bands and if the serum CC16 concentration was more than 9 ng/ml, the assay produced three bands at the test zone. One control band is observed at the control line irrespective of the CC16 concentration present in the serum (Fig. 1). Clinical significance of the results obtained in terms of the number of bands visualized and their relation with CC16 concentration is depicted in Table 1 and Fig. 1 below.

**Table 1:** Details of samples tested by ELISA and its comparison with lateral flow assay with the classification according to three categories of detection.

Sr. No	Clinical significance	CC16 value range by ELISA	No. of samples	Number of bands at test line	Matching	Un-matching
1	Suspected moderate to severe silicosis	6 ng/ml or less	34	1	33	1
2	Suspected early silicosis	6.1–9 ng/ml	34	2	34	0
3	Healthy or very early stage of silicosis that usually remains undetectable by chest x-ray	9.1 ng/ml or high	38	3	36	2

### Performance evaluation of the assay

The serum samples were tested in parallel by commercially available ELISA kit and the semi-quantitative lateral flow assay. The comparative evaluation was performed by estimating CC16 concentration by ELISA and numbers of bands on the lateral flow assay (Table 1). The comparative evaluation of ELISA and lateral flow strip test showed that out of 106 serum samples, 34 samples were in the range of 0–6 ng/ml CC16 concentration. Thirty-three samples exhibited 1 band at the test zone and one band at the control line. While one sample with CC16 concentration of 5.034 exhibited two bands at the test zone. In the range of 6.1–9 ng/ml concentration of CC16, there were 34 samples. These samples exhibited two bands at the test zone and one band at the control line. In the range of 9.1 and above concentration of CC16 there were 38 samples. Thirty-six out of 38 samples exhibited 3 bands at the test zone and one band at the control line. While the rest of the two samples exhibited two bands at the test zone. The details are mentioned in Table 2. The results of performance evaluation of semi-quantitative lateral flow assay with ELISA using 106 serum samples have been summarized in Table 2.

**Table 2:** Performance evaluation of lateral flow assay for semi-quantification of CC16 in 104 serum samples in comparison with ELISA.

CC16 concentration ng/ml	Nos. samples	Nos. of bands on the strip			
		NIL	ONE	TWO	THREE
< = 1	1	1	0	0	0
1.1–2	0	0	0	0	0
2.1–3	8	0	8	0	0
3.1–4	7	0	7	0	0
4.1–5	12	0	12	0	0
5.1–6	6	0	5	1	0
6.1–7	8	0	0	8	0
7.1–8	17	0	0	17	0
8.1–9	9	0	0	9	0
9.1–10	4	0	0	2	2
10.1–11	6	0	0	0	6
11.1–12	2	0	0	0	2
12.1–14	3	0	0	0	3
14.1–16	7	0	0	0	7
> 16	16	0	0	0	16
Total	106	1	32	37	36

NB—only three samples were discordant (two had > 9 ng/ml and one had < 6 ng/ml) and did not match with the expected ELISA results.

It may be noted that the gray zone was observed with very few of our test samples having a CC16 concentration of 9–9.5 ng/ml and 5 to 6 ng/ml. There were four samples in the range 9–9.5 ng/ml of CC16 concentrations. Out of four, two samples exhibited two bands at the test zone and two samples exhibited three bands at the test zone. While in the range of 5 to 6 ng/ml CC16 concentration, there were 6 samples. Out of six samples, one sample having CC16 concentration 5.034 ng/ml displayed two bands at the test zone (Table 2). It is understood that for any biological event, there may be some variation or overlapping of values at the cut off end or marginal region. Hence, above is an expected event and may be ignored considering it as a normal biological phenomenon.

### Statistical analysis

The performance evaluation of the semi-quantitative lateral flow assay was performed considering the samples with CC16 concentration > 9 ng/ml as negative for silicosis and < 9 ng/ml as positive for silicosis. The sensitivity of the assay was 100% while the specificity was 95%.

## Discussion

Earlier study conducted by ICMR-National Institute of Occupational Health (ICMR-NIOH), Ahmedabad, had conclusively evidenced that serum CC16 might be a useful proxy marker for early detection of silicosis among silica dust exposed workers. Subsequently ICMR-NIOH study showed that when serum CC16 value ranges between > 6 and 9 ng/ml, it is indicative of early silicosis, which needs to be confirmed by chest X-ray or CT scan for further necessary action including notification, clinical management and compensation^21,23^. Hence, a semi-quantitative kit has been developed by ICMR that would detect the suspected early silicosis among silica dust exposed workers. This kit is intended for periodic screening (preferably annual or bi-annual if silica content of the inhaled dust is high) among silica dust exposed workers for early diagnosis and prevention/control of silicosis and to be managed by the network of peripheral health care workers of the entire country. Necessary logistic support and training of primary health workers are essential components towards this silicosis control initiative.

The principle of this assay is that the serum CC16 protein migrates by capillary action through the nitrocellulose membrane and reaches the test lines. At the test line a certain amount of serum CC16 is captured by the rabbit anti CC16 polyclonal antibodies. The excess amount of CC16 protein is trapped by the second test line and the same is followed by the third test line. This is followed by running GNPs conjugated with anti-CC16 monoclonal antibodies (anti CC16 mAb + GNPs complex) through the NCM which will produce a red colored band at the test line and the control line. The intensity and the number of red lines developed at the CRP test zone were directly proportional to the concentration of CC16 in the sample semi quantitatively. This in turn could be used to predict the suspected degree of silicosis (extent of lung damage) in the patients.

Interpretation of the results was done by checking the control line. The control line is indicative of valid reaction. If the control line does not appear, the reaction is invalid. If the serum CC16 concentration is 6 ng/ml or less, one red colored band is detected. If the serum CC16 concentration is in the range of 6.1 to 9 ng/ml then the assay produces two bands and if the serum CC16 concentration is more than 9 ng/ml, the assay produces three bands. One control band is observed at the control line irrespective of the CC16 concentration present in the serum.

The performance of the assay was evaluated by testing 106 serum samples (68 silica exposed workers and 38 healthy individuals). The comparative evaluation was performed by estimating CC16 concentration by ELISA and numbers of bands on lateral flow assay. The comparative evaluation of ELISA and lateral flow strip test showed that out of 106 serum samples 34 samples were in the range of 0–6 ng/ml of CC16 concentration. Thirty-three out of thirty-four samples exhibited 1 band at the test zone and one band at the control line. The patients having 0–6 ng/ml of CC16 concentration are suspected to have either moderate (3.1–6 ng/ml) or advanced (0 to 3 ng/ml) silicotic lung injury. In the range of 6.1–9 ng/ml concentration of CC16, there were 34 samples. These samples exhibited two bands at the test zone and one band at the control line. The patients having serum CC16 value of 6.1 to 9 ng/ml are suspected to have a mild or early silicosis that is detectable by chest x-ray/CT scan for confirmation. Since smoking usually reduces mean serum CC16 value by 1–2 ng/ml compared to non-smokers, 6.1 to 9 ng/ml will include the value range for early silicosis both in non-smoking as well as smoking workers, considering a cut off value of 9 ng/ml or slightly less for non-smokers and 1–2 ng/ml less than the above-said value for smokers. Hence, the range of 6.1–9 ng/ml is expected to include both smokers and non-smokers together for operational ease in a public health program point of view.

In the range of 9.1 and above, there were 38 samples. Thirty-six out of thirty-eight serum samples exhibited 3 bands at the test zone and one band at the control line. The patients having serum value of more than 9 ng/ml of CC16 but up to 12 ng/ml (with occupational silica dust exposure history) may be in their very early pathological process of silicosis which will not cast shadow by chest x-ray. Values above 12 ng/ml are usually healthy individuals. The present study has considered two issues for diagnosis of silicosis at an early stage – serum CC16 value by screening among silica dust exposed workers followed by chest x-ray/CT scan for confirmation. Considering above, the range of 6.1–9 ng/ml appears to be useful for early detection range for serving triple purposes – better clinical management including minimizing further exposure, notification & compensation.

In conclusion, it may be said that this Point of Care kit may be employed for semi-quantitative estimation of CC16 in human serum samples for the selective population (having a history of occupational silica dust exposure). The inverse relationship between serum CC16 levels and the severity of silicosis has already been evidenced in the previous studies performed by ICMR-NIOH^21^. The purpose of this paper is to demonstrate the performance evaluation of LFA in comparison with ELISA to validate the developed assay. The purpose of this manuscript is to provide an assay for the screening of the silica dust exposed workers. The utility of this screening assay is to identify the individuals for further medical interventions. The individuals exhibiting 2 or less number of bands on LFA (CC16 concentration < 9 ng/ml) are may be in the early stages of silicosis and should be advised thorough medical examination such as spirometry, chest X-ray or CT scan etc. for confirmation. The individuals exhibiting three bands in LFA (CC16 concentration > 9 ng/ml) need not go for X-ray. This can significantly decrease the risk of X-ray exposure to the individuals. Hence, this assay would be useful for early detection of silicosis for various purposes such as notification to the local authority, secondary prevention and financial compensation as per guidelines of the country.

## Methods

The principle of this assay is that the serum CC16 protein migrates by capillary action through the nitrocellulose membrane and reaches the test lines. At the first test line a certain amount of serum CC16 is captured by the rabbit anti CC16 polyclonal antibodies. The excess amount of CC16 protein is trapped by the second test line followed by the third test line. This is followed by running GNPs conjugated with anti-CC16 monoclonal antibodies (anti CC16 mAb + GNPs complex) through the NCM which will produce a red colored band at the test line and the control line. The intensity and the number of red lines developed at the CRP test zone were directly proportional to the concentration of CC16 in the sample semi quantitatively. This in turn can be used to predict the suspected degree of silicosis (lung damage) in the patients.

### Raw materials

In the present study, the lateral flow membranes were purchased from MDI Membrane technologies Ambala, India. There are many types of membranes available out of them CNPF-SN12-L2-P25 10 µm membrane was used in this study. The CNPF-SN12 membrane is associated with lower protein binding. The L2 means Laminate with NC membrane mounted on it and adhesive placed for sample pad and absorbent pad. The 10 µm is the porosity of the nitrocellulose (NC) membrane.

The antibodies used in this study were procured from commercial sources. The antibody used for coating the membrane was rabbit anti human CC16 polyclonal antibody and it was purchased from (Catalogue No. 500-P330) PeproTech, USA. The antibody used for conjugation with gold nanoparticles (GNPs) was anti-CC16 monoclonal antibody and it was purchased from (Catalogue No. MA1-40,223) Thermo Fisher scientific, USA.

The recombinant protein produced in *E. coli* was used as the standard in this assay. The recombinant club cell protein was a 9.2 kDa size consisting of 80 amino acids. This was purchased from (Catalogue No. RD191022200) Biovendor – Laboratorni medicina a.s. Czech Republic.

The colloidal spherical gold nanoparticles were purchased from (Catalogue No. 741981-25ML) Sigma Aldrich USA.

### Samples collection

Sample size: Assuming the sensitivity of the assay as 95%, prevalence of silicosis as 69.1%^24^ at 5% level of significance, and absolute precision as 5% a total 106 samples were needed^25^.

A total of 106 samples were collected for this study. The samples were collected by ICMR-NIOH from occupational health clinic in Delhi and were transported to ICMR-NIV, Mumbai unit. The 38 samples were collected from healthy individuals as a control group and 68 were collected from silica exposed individuals confirmed by chest X-ray. About 3 ml blood sample was collected by venipuncture using a vacutainer from each eligible and consented participant. The blood samples were allowed to coagulate by keeping the collection tubes in a slant manner. After coagulation of blood, the tubes were centrifuged at 1500 rpm for 5 min to separate the serum. The serum was separated in cryo-vials and stored at -20 °C till further use.

### ELISA for the quantitation of CC16

The CC16 concentrations in serum samples were determined using a commercially available ELISA kit (Catalogue No. RD191022200 Biovendor – Laboratorni medicina a.s. Czech Republic). This ELISA was used as the reference method for comparative evaluation of the semi-quantitative lateral flow assay. This is an antigen capture type ELISA which is also known as antigen sandwich ELISA. The ELISA was performed as per instruction manual provided by the kit and a brief description of the protocol is as follows.

The kit consists of a pre-coated ELISA strip in which anti-CC16 polyclonal antibody is coated. The serum samples as well as the standard protein samples were diluted 25 times (5 µl serum in 120 µl dilution buffer) and allowed to react with the pre-coated ELISA strips for 60 min at room temperature with shaking at 300 rpm followed by a quick wash. Then the biotin labelled polyclonal anti-human club cell protein antibody was added and incubated with captured club cell protein for 60 min. After another washing, streptavidin–horseradish peroxidase conjugate was added. After 60-min incubation and the last washing step, the remaining conjugate was allowed to react with the substrate solution (TMB). The reaction was stopped by addition of acidic solution and the absorbance of the resulting yellow product was measured using the TECAN M200 ELISA plate reader.

### Conjugation of antibody with Gold Nanoparticles (GNPs)

A volume of 2 ml of mono dispersed GNPs solution (40 nm, negative charge) was taken in two separate sterile 1.5 ml tubes (1 ml in each tube). GNPs solution was centrifuged at 13,200 rpm at 4 °C for 5 min. 500 µl of supernatant was discarded from each tube and the soft pellets were resuspended in the remaining solutions.

Contents of the two tubes were pooled together. The pH of the pooled GNPs solution was adjusted to 9 using 0.1 M K_2_CO_3_ solution. Generally, 12–15 µl of 0.1 M K_2_CO_3_ solution is enough to adjust the pH to 9 for 1 ml GNPs solution. 10 µg of anti-CC16 mAb in 10 mM Tris HCl (pH 8) was added to the above prepared 1 ml GNPs solution drop wise with gentle mixing by inverting 5 times.

The tube was incubated at room temperature for 10 min. Bovine serum albumin (BSA) at a final concentration of 0.025% (W/V) was dissolved in 10 mM Tris HCl (pH 8). Said buffer was added drop wise for blocking unoccupied sites on the GNPs mixed by inverting the tube 5 times. The tube was incubated at room temperature for 10 min. The reaction mixture was centrifuged at 4000Xg for 1 h at 4 °C.

Maximum possible supernatant was discarded carefully without disturbing the soft pellet. The soft pellet was resuspended in 50 µl of 10 mM Tris–HCl pH 8 with 0.1% (w/v) BSA. The conjugate containing tube was covered with aluminum foil and stored between 2 and 8 °C until further use. The working solution of the conjugate was diluted 1:5 times (1 µl conjugate + 4 µl of 10 mM Tris–HCl pH 8 with 0.1% (w/v) BSA buffer containing tween-20).

### Lateral flow assay (LFA)

The CNPF-SN12-L2-P25 10 µm membrane strips were used for this assay. Each strip having 0.5 cm width contains a 2.5 cm patch of nitrocellulose membrane (NCM) for the reaction.

**Figure 2:**
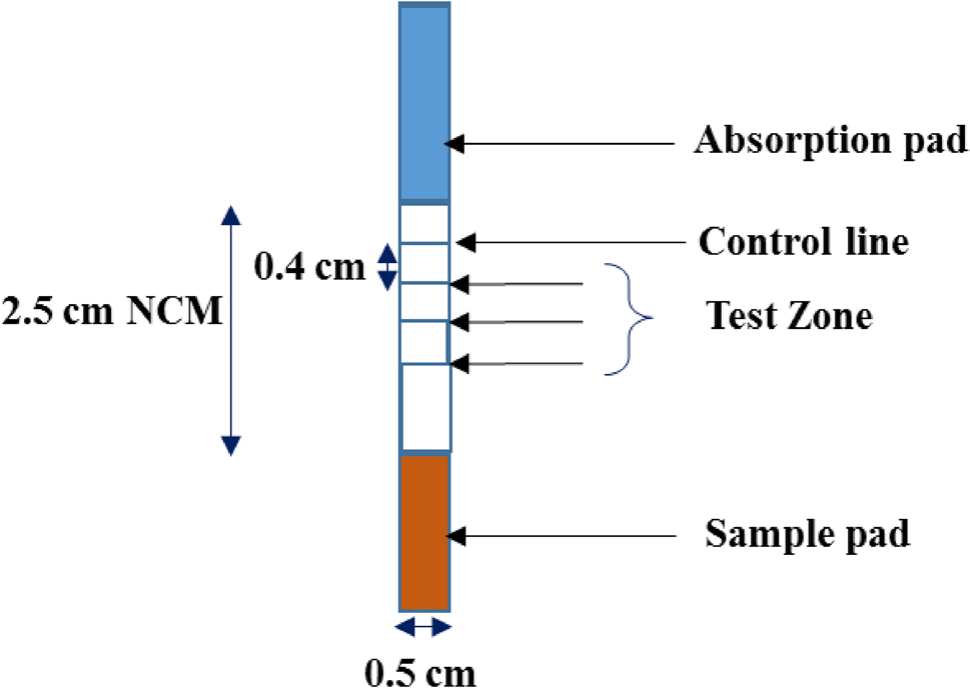
Schematic representation of lateral flow assay strip preparation. The figure describes a single strip of LFA with the width of 0.5 cm; length of NCM 2.5 cm and location of control line at the top and three test lines 0.4 cm below subsequently.

The lateral flow strip was coated with three test lines and one control line on the NCM strip. The control line was located at the top (0.4 cm from the edge of NCM) and below that, there were three test lines located 0.4 cm from each other (Fig. 2). The control line was coated with the goat anti-mouse antibody 0.2 µg/µl in 10 mM Tris HCl buffer pH 8. The three test lines were coated with a specific capture antibody (Rabbit anti CC16 polyclonal antibody) in 10 mM Tris HCl buffer pH 8. After coating, the strips were allowed to dry at 37 °C for 1 h. After drying the absorption pad was attached at the far end of the test strip in the direction of flow 1 mm overlapping the NCM.

### Sample application and testing

The strips were placed at a horizontal surface.10 µl of each serum sample was loaded on each strip and allowed to flow through the membrane. 10 µl of working GNPs + anti-CC16 mAb conjugate was loaded and allowed to flow through the membrane strip. Again 10 µl of the wash buffer was allowed to flow through the membrane to wash off the extra GNPs + anti-CC16 mAb conjugate from the strip. Then the number of bands were observed on the lateral flow strip. The interpretation of the results was done by checking the control line. The control line was indicative of valid reaction. If the control line does not appear, the reaction is invalid. The intensity and the number of red lines developed at the CRP test zone were directly proportional to the concentration of CC16 in the serum samples.

### Performance evaluation of lateral flow assay

The performance of the assay was evaluated by testing 106 serum samples. The serum samples were also tested by a commercially available ELISA kit. The comparative evaluation was performed by estimating CC16 concentration by ELISA and numbers of bands on lateral flow assay. The statistical analysis was performed to calculate the sensitivity and specificity.

### Ethics statements

The study was approved by the Institute Ethics Committee (IEC) of Indian Council of Medical Research – National Institute of Occupational Health (ICMR-NIOH), Ahmedabad, India. The authors confirm that all methods were performed in accordance with the relevant guidelines and regulations of declaration of Helsinki of ethical principles for medical research involving human subjects. A written informed consent was obtained from all eligible participants voluntarily before initiating this study.
